# Human Neonatal Rotavirus Vaccine (RV3-BB) Produces Vaccine Take Irrespective of Histo-Blood Group Antigen Status

**DOI:** 10.1093/infdis/jiz333

**Published:** 2019-11-25

**Authors:** Karen Boniface, Sean G Byars, Daniel Cowley, Carl D Kirkwood, Julie E Bines

**Affiliations:** 1 Enteric Diseases Group, Murdoch Children’s Research Institute, Seattle, Washington; 2 Melbourne School of Population and Global Health, Seattle, Washington; 3 Department of Pediatrics, University of Melbourne, Seattle, Washington; 4 Bill and Melinda Gates Foundation, Seattle, Washington; 5 Department of Gastroenterology and Clinical Nutrition, Royal Children’s Hospital, Parkville, Australia

**Keywords:** Rotavirus, RV3-BB, histo-blood group antigens, neonatal vaccination, vaccine take, Lewis antibodies, secretor status

## Abstract

**Background:**

VP4 [P] genotype binding specificities of rotaviruses and differential expression of histo-blood group antigens (HBGAs) between populations may contribute to reduced efficacy against severe rotavirus disease. P[6]-based rotavirus vaccines could broaden protection in such settings, particularly in Africa, where the Lewis-negative phenotype and P[6] rotavirus strains are common.

**Methods:**

The association between HBGA status and G3P[6] rotavirus vaccine (RV3-BB) take was investigated in a phase 2A study of RV3-BB vaccine involving 46 individuals in Dunedin, New Zealand, during 2012–2014. *FUT2* and *FUT3* genotypes were determined from DNA extracted from stool specimens, and frequencies of positive cumulative vaccine take, defined as an RV3-BB serum immune response (either immunoglobulin A or serum neutralizing antibody) and/or stool excretion of the vaccine strain, stratified by HBGA status were determined.

**Results:**

RV3-BB produced positive cumulative vaccine take in 29 of 32 individuals (91%) who expressed a functional FUT2 enzyme (the secretor group), 13 of 13 (100%) who were *FUT2* null (the nonsecretor group), and 1 of 1 with reduced FUT2 activity (i.e., a weak secretor); in 37 of 40 individuals (93%) who expressed a functional FUT3 enzyme (the Lewis-positive group) and 3 of 3 who were *FUT3* null (the Lewis-negative group); and in 25 of 28 Lewis-positive secretors (89%), 12 of 12 Lewis-positive nonsecretors (100%), 2 of 2 Lewis-negative secretors, and 1 of 1 Lewis-negative weak secretor.

**Conclusions:**

RV3-BB produced positive cumulative vaccine take irrespective of HBGA status. RV3-BB has the potential to provide an improved level of protection in settings where P[6] rotavirus disease is endemic, irrespective of the HBGA profile of the population.

Rotavirus vaccines have made a major impact on rotavirus hospitalizations and death due to rotavirus gastroenteritis. More than 98 countries have now introduced rotavirus vaccines nationally or subnationally within their national immunization programs. Despite this success, both Rotarix and RotaTeq vaccines have reduced efficacy against severe rotavirus disease in low-income countries (49.2%–72.2% efficacy for Rotarix and 64.2% efficacy for RotaTeq), compared with high-income countries (>88% efficacy for each vaccine) [1–4]. The genotypic diversity of rotavirus is greater in Africa, compared with other continents. In sub-Saharan Africa, there is an increased proportion of P[6] rotavirus strains causing severe disease; P[6] strains accounted for 22.6% of rotaviruses causing disease between 2006 and 2016, whereas they have been detected only sporadically in higher-income countries globally [5–7]. Population differences in histo-blood group antigen (HBGA) status could contribute to these differences in genotypes circulating within a population and the level of protection provided by vaccines based on P[8] strains.

HBGAs are neutrally charged carbohydrates that are expressed in humans on red blood cells; on the mucosal epithelia of the digestive, respiratory, and genitourinary tracts; and, in soluble form, in secretions such as saliva and breast milk. Enteric pathogens frequently use HBGAs as the first step in the cell attachment and entry process and, consequently, can be key determinants of a pathogen’s host range and tissue tropism [8–11]. For rotavirus, infectivity is mediated by trypsin cleavage of the VP4 outer capsid protein to produce the VP8* and VP5* subunits; initial attachment to host cells is mediated by VP8*, whereas VP5* is required for cellular entry [12, 13]. The binding of the VP8* of some human rotavirus strains to HBGAs has been demonstrated, strongly suggesting that HBGAs are important host factors or cellular receptors [14–18].

The biosynthesis of type 1 HBGAs occurs by the stepwise addition of monosaccharide units to precursor disaccharide molecules. This process is catalyzed by the enzymes fucosyltransferase 2 (FUT2) and fucosyltransferase 3 (FUT3), encoded by *FUT2* and *FUT3*, respectively. Both genes have dominant alleles (Se for *FUT2* and Le for *FUT3*) that encode functional enzymes, and recessive alleles (se and le, respectively), caused by specific single-nucleotide polymorphisms (SNPs), that do not code functional enzymes. Individuals who express a functional FUT2 enzyme are referred to as “secretors” and have the genotype Se/Se or Se/se, whereas those who are *FUT2* null are known as “nonsecretors” and have the genotype se/se. Similarly, individuals who express a functional FUT3 enzyme are categorized as “Lewis positive” and have the genotype Le/Le or Le/le, whereas *FUT3* null individuals are classified as “Lewis negative” and have the genotype le/le [19].

The HBGA phenotype of an individual represents the different combinations of the presence or absence of functional FUT2 and FUT3 enzymes and can be determined by assaying for the resulting antigens that are detectable in secretions. Lewis-positive secretors have functional FUT2 and FUT3 and produce Le^b^ antigen, Lewis-positive nonsecretors have functional FUT3 but not FUT2 and produce Le^a^ antigen, and Lewis-negative secretors have functional FUT2 but not FUT3 and produce H type 1 antigen; in Lewis-negative nonsecretors, neither FUT2 nor FUT3 is functional, and thus Le^b^, Le^a^, and H type 1 antigens are not produced. The prevalence of the HBGA phenotypes varies between populations; approximately 75% of Europeans, 50%–60% of Africans, and 42% of Asians are Lewis-positive secretors, whereas only 20% of Europeans are Lewis-positive nonsecretors. The Lewis-negative phenotype is less common in Europeans and Asians (8% and 7%, respectively), whereas it was detected at a higher rate (32%) in Burkina Faso in West Africa [20–22]. The phenotype for individuals categorized as “weak secretors,” in which the enzyme activity of FUT2 is decreased because of a specific missense mutation at nucleotide position 385 (A > T), occurs in 10%–20% of Southeast and East Asian populations [23, 24].

The Lewis and secretor status of an individual may mediate susceptibility to rotavirus infection, including vaccination with a live viral vaccine, as the binding specificity of rotavirus to HBGAs may be VP4 [P] genotype dependent. For P[8] rotavirus strains, including the P[8]-based Rotarix vaccine, secretors have been observed to be more susceptible to infection and vaccine take than nonsecretors. vaccine take than nonsecretors [25–33]. The role of Lewis status is less clear, but the Lewis-negative phenotype was more common in infants who developed P[6] rotavirus gastroenteritis following a full 2-dose course of Rotarix, compared with community controls (odds ratio, 3.2; 95% confidence interval, 1.4–7.2) [28]. For P[6] rotaviruses, limited epidemiological studies and in vitro binding assays have demonstrated differential HBGA receptor specificity when compared to P[8] and P[4] strains [34]. The VP4 [P] genotype–dependent binding specificity of rotaviruses and the differential expression of HBGAs between populations could contribute to the reduced efficacy against severe rotavirus disease for Rotarix and RotaTeq observed in low-income settings with a high burden of rotavirus disease. It is plausible that a P[6]-based rotavirus vaccine could play an important role in broadening protection in Africa, where the Lewis-negative phenotype is more prevalent and where P[6] rotavirus strains are endemic.

The RV3-BB human neonatal rotavirus vaccine is based on an isolate of a G3P[6] human neonatal rotavirus strain that circulated among healthy newborns in obstetric hospitals in Melbourne, Australia [35]. A phase 2A double-blinded, randomized, placebo-controlled, single-center, 3-arm parallel group study of oral RV3-BB rotavirus vaccine was conducted at a single center in Dunedin, New Zealand, between 13 January 2012 and 17 April 2014, which has been previously described (Australian New Zealand Clinical Trials Registry identifier ACTRN12611001212943) [36]. Vaccine take was demonstrated in >90% of participants in this trial after administration of 3 doses of RV3-BB vaccine, when the first dose was administered 0–5 days after birth (neonatal schedule) or at approximately 8 weeks of age (infant schedule). The aim of the current study was to determine whether Lewis and secretor status influenced vaccine take after vaccination with the G3P[6] human neonatal rotavirus vaccine RV3-BB.

## METHODS

### Subjects and Samples

**Figure 1.**
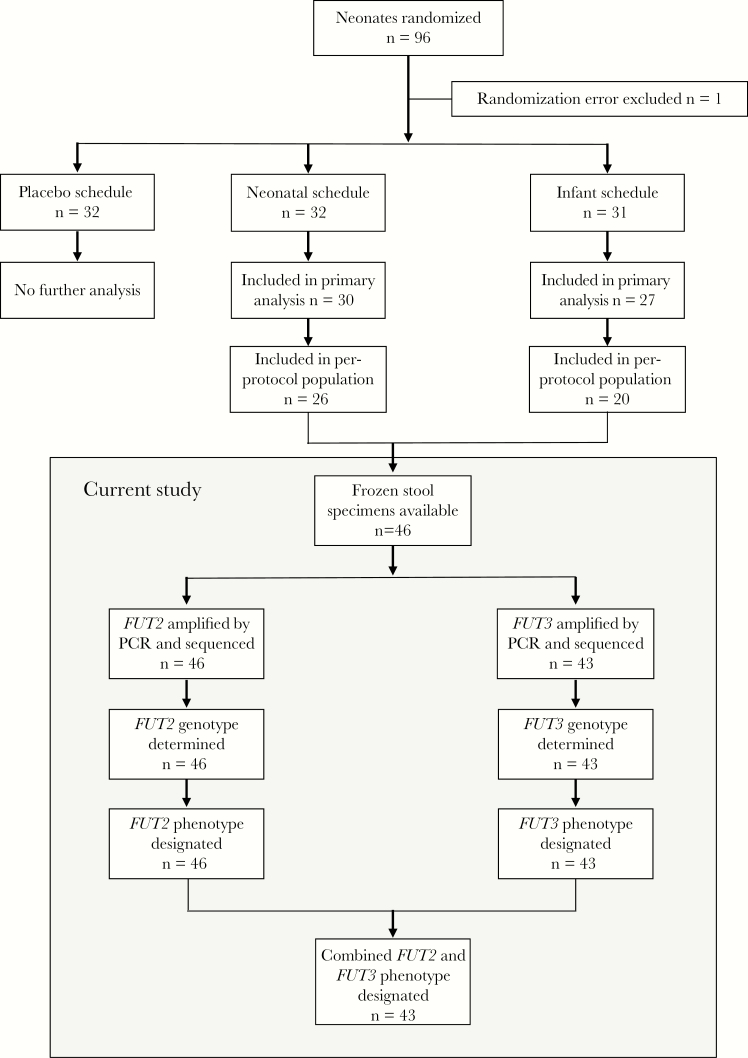
Flow of participants through the study selection process and summary of stool specimen analyses. PCR, polymerase chain reaction. *FUT2*, gene encoding fucosyltransferase 2; *FUT3*, gene encoding fucosyltransferase 3.

This study was performed on frozen stool samples collected from all participants in the per protocol population in the phase 2A trial in New Zealand for whom written informed consent was provided by the parent(s)/guardian for future evaluation of studies of rotavirus (n = 46; Figure 1). The protocol was approved by the Lower South Region Ethics Committee, New Zealand; the Human Research Ethics Committee, Royal Children’s Hospital, Australia; and the New Zealand Medicines and Medical Devices Safety Authority.

### DNA Extraction From Frozen Stool Specimens

DNA was extracted from a 200-mg stool specimen from each participant, using the NucleoSpin DNA Stool kit (Macherey-Nagel, Düren, Germany) according to the manufacturer’s instructions, with the following modifications. For the disruption and homogenization step, samples were agitated for 30 seconds and rested for 1 minute, for 4 rounds, at room temperature, using the Mini-Beadbeater (Biospec Products, Bartlesville, OK). An intermediate volume of elution buffer (100 µL) was used to elute DNA, to dilute residual polymerase chain reaction (PCR) inhibitors such as bile salts and complex polysaccharides that are inherent to stool. Eluted DNA was stored at −30°C.

### *FUT2* and *FUT3* Amplification

A 1184-bp region and a 1491-bp region spanning the enzyme-coding regions of *FUT2* and *FUT3*, respectively, were amplified by PCR. The *FUT2* primers were 5′-CTAACGTGTCCCGTTTTCCTC-3′ (forward) and 5′-CCCAACGCATCTTCACAGA-3′ (reverse). The *FUT3* primers were 5′-GGAGCTTTGGTAAGCAGGAG-3′ (forward) and 5′-TCAGTGTGGCAAGGTCTCTG-3′ (reverse). The forward primer sequences have been described previously [33, 37], and reverse primers were designed for this project. Lyophilized high-performance liquid chromatography–purified primers (Merck, Darmstadt, Germany) were resuspended to 10-µM working stocks, using UltraPure DNase/RNase-free distilled water (catalog no. 10977-015; Invitrogen).

### PCR Amplification and Complementary DNA Purification

PCR amplification was performed for each amplicon, using the PrimeSTAR GXL DNA polymerase kit (Takara, Kusatsu, Japan), according to manufacturer’s instructions. Briefly, 50 µL PCR reactions were prepared, with each containing nuclease-free water, 1× PrimeSTAR GXL buffer with 5 mM MgCl_2_, 200 µM dNTPs, 0.2 µM each of the forward and reverse primers, 1.25 U of PrimeSTAR GXL DNA polymerase, and 5 µL of genomic DNA. PCR was performed as described previously [33]: 1 cycle at 94°C for 2 minutes; 25 cycles at 94°C for 30 seconds, at 65°C for 30 seconds, and at 72°C for 90 seconds; and 20 cycles at 94°C for 30 seconds, at 55°C for 30 seconds, and at 72°C for 90 seconds). Amplicons were purified using the QIAquick Gel Extraction Kit (Qiagen, Hilden, Germany).

### *FUT2* and *FUT3* Genotyping

The nucleotide sequence of purified *FUT2* and *FUT3* amplicons was determined by next-generation sequencing (NGS; Centre for Genomics Medicine—Sequencing Service and Development Platform, Victorian Clinical Genetic Services/Murdoch Children’s Research Institute). In brief, complementary DNA was amplified and purified with AMpure magnetic beads (Beckman Coulter), and libraries for NGS were then prepared using the Nextera XT library prep kit (Illumina). Libraries were pooled in equimolar ratios and sequenced on the MiSeq system (Illumina), using 2 × 150-bp sequencing.

Before alignment, paired-end fastq files were initially screened for potential quality problems with FastQC, version 11.8, and the human reference (genome) assembly GRCh38 was indexed with SAMtools, version 1.5. Alignment of reads to the reference genome was performed using the Burrows-Wheeler Alignment tool, version 7.15, with the *mem* algorithm. Aligned reads were then prepared for SNP calling by converting SAM files to BAM files and were sorted with SAMtools. SNPs were then called with the SAMtools mpileup function and the BCFtools, version 1.5, call function. SNPs were then filtered and annotated with BCFtools.

### Inferring Lewis and Secretor Status

The Lewis and secretor phenotype for each participant was designated by analysis of the SNPs identified in the coding regions of each gene. For *FUT2*, participants who were homozygous mutants for the G428A (rs601338) nonsense and G739A (rs602662) missense SNPs were designated as nonsecretors, and those who were homozygous mutants at the A385T (rs1047781) missense SNP were designated as weak secretors. Conversely, those who were wild type or heterozygous mutants for these SNPs were designated as secretors. For *FUT3*, participants who were homozygous mutants for at least one of the T202C (rs812936), G508A (rs3745635) or T1067A (rs3894326) missense SNPs were designated as Lewis negative. Conversely, those that were wild-type or heterozygous mutants for these SNPs were designated as Lewis positive. Participants who were homozygous mutants for the T59G (rs28362459) reducing or the C314T (rs778986) missense SNPs were designated Lewis negative only if these mutations were seen in addition to the T202C, G508A or T1067A missense SNPs.

### Statistical Analyses

Frequencies of positive cumulative vaccine take by Lewis, secretor and combined Lewis and secretor status were expressed as proportions (and percentages). Cumulative vaccine take was defined as a serum immune response (ie, a ≥3-fold increase in titer from baseline) of anti-rotavirus immunoglobulin A (IgA) or serum neutralizing antibodies 28 days following dose administration or as detection of RV3-BB virus excretion by reverse transcription PCR analysis of stool specimens at least once during days 3–7 following dose administration, as previously described [36]. χ ^2^ analysis and relative risks (RRs) were used to compare frequencies of vaccine take between variables, using Stata, version 15.1 (StataCorp, College Station, TX). Differences were considered statistically significant at a *P* value of < .05.

## RESULTS

The demographic characteristics of the study subset were similar to those previously described for the intention-to-treat cohort: age at first dose of investigational product, sex, race, gestational age, and birth weight (Supplementary Table 1) [36]. DNA was extracted from stool specimens from all 46 participants, and *FUT2* and *FUT3* were amplified by PCR for 46 of 46 and 43 of 46, respectively. Amplicons for both genes were obtained for 43 of 46 participants. The nucleotide sequence was determined by NGS, and SNPs were called on the basis of alignment with a human reference genome.

### *FUT2* and *FUT3* Genotypes and Inferring Phenotypes

For *FUT2*, 13 participants (28%) were homozygous mutants for both the nonsense variant G428A (rs601338) and the missense variant G739A (rs602662) and were designated as nonsecretors (Table 1). One participant (2%) was a homozygous mutant for the missense variant A385T (rs1047781) and was designated as a weak secretor. The remaining 32 participants (70%) were either wild type or heterozygous mutants for these SNPs and were designated as secretors. Supplementary Table 2 shows the distribution of *FUT2* SNPs and the allele frequencies detected in the cohort, and Supplementary Table 3 shows *FUT2* genotypes and phenotype designations for each participant.

**Table 1.** Distribution of Lewis and Secretor Phenotypes

Phenotype	Participants, No. (%)
Secretor phenotype (n = 46)	
Secretor	32 (70)
Nonsecretor	13 (28)
Weak secretor	1 (2)
Not determined	0
Lewis phenotype (n = 46)	
Positive	40 (86)
Negative	3 (7)
Not determined	3 (7)
Combined phenotype (n = 46)	
Le, Se (Lewis positive, secretor)	28 (61)
Le, se (Lewis positive, nonsecretor)	12 (26)
le, Se (Lewis negative, secretor)	2 (4)
le, se^w^ (Lewis negative, weak secretor)	1 (2)
le, se (Lewis negative, nonsecretor)	0
Not determined	3 (7)

Phenotypes were inferred from genotype type data. See “Methods” for descriptions of secretor and Lewis phenotypes.

Abbreviations: le, nonfunctional FUT3; Le, at least 1 functional gene encoding FUT3; RR, relative risk; se, nonfunctional FUT2; Se, at least 1 functional gene encoding FUT2.

For *FUT3*, 3 participants (7%) were designated as Lewis negative (Table 1). Of these, 1 was a homozygous mutant for the T202C (rs812936) and C314T (rs778986) missense variants, 1 was a homozygous mutant for the T59G (rs28362459) reducing and G508A (rs3745635) missense variants, and 1 was homozygous mutant for the T59G (rs28362459) reducing and T1067A (rs3894326) missense variants. Forty participants (86%) were either wild type or heterozygous mutants for these SNPs and were designated as Lewis positive. Lewis phenotype was not designated for 3 participants, owing to failure of *FUT3* amplification by PCR. Supplementary Table 4 shows the distribution of *FUT3* SNPs and the allele frequencies detected in the cohort, and Supplementary Table 5 shows *FUT3* genotypes and phenotype designations for each participant.

Overall, 28 participants (61%) were designated as Lewis-positive secretors, 12 (26%) as Lewis-positive nonsecretors, 2 (4%) as Lewis-negative secretors, and 1 (2%) as a Lewis-negative weak secretor (Table 1). There were no Lewis-negative nonsecretors in the cohort.

### Vaccine Take, by Lewis and Secretor Status

RV3-BB produced positive cumulative vaccine take, irrespective of the secretor, Lewis, and combined Lewis and secretor status of participants in the cohort (Table 2). Vaccine take was detected in 29 of 32 secretors (91%), 13 of 13 nonsecretors (100%), and 1 of 1 weak secretor (100%); 37 of 40 Lewis-positive participants (93%) and 3 of 3 Lewis-negative participants (100%); and 25 of 28 Lewis-positive secretors (89%), 12 of 12 Lewis-positive nonsecretors (100%), 2 of 2 Lewis-negative secretors (100%), and 1 of 1 Lewis-negative weak secretor (100%). There were no Lewis-negative nonsecretors in the cohort, so vaccine take could not be assessed for this phenotype.

**Table 2:** Proportion (%) Positive Vaccine Take by Lewis and Secretor Status

	Positive Vaccine Take			Positive for Serum IgA and/or SNA Response			Positive for Serum IgA Response			Positive for SNA Response			Positive for RV3-BB Virus Excretion		
Phenotype^a^	Participants, Proportion (%)	RR (95% CI)	*P*	Participants, Proportion (%)	RR (95% CI)	*P*	Participants, Proportion (%)	RR (95% CI)	*P*	Participants, Proportion (%)	RR (95% CI)	*P*	Participants, Proportion (%)	RR (95% CI)	*P*
Secretor phenotype (n = 46)															
Secretor	29/32 (91)	Reference		21/32 (66)	Reference		21/32 (66)	Reference		4/31 (13)	Reference		23/32 (72)	Reference	
Nonsecretor	13/13 (100)	0.91 (.81–1.01)	.25	11/13 (85)	0.76 (.55–1.09)	.20	11/13 (85)	0.78 (.55–1.09)	.20	3/11 (27)	0.47 (.13–1.79)	.27	10/13 (77)	0.93 (.65–1.35)	.73
Weak secretor	1/1 (100)	…		1/1 (100)	…		1/1 (100)	…		0/1 (0)	…		1/1 (100)	…	
Lewis phenotype (n = 43)															
Positive	37/40 (93)	Reference		29/40 (73)	…		29/40 (73)	…		7/38 (18)	…		28/40 (70)	…	
Negative	3/3 (100)	0.93 (.85–1.01)	.62	1/3 (33)	…		1/3 (33)	…		0/3 (0)	…		3/3 (100)	…	
Combined phenotype (n = 43)															
Le, Se (Lewis positive, secretor)	25/28 (89)	Reference		19/28 (68)	Reference		19/28 (68)	Reference		4/27 (15)^j^	Reference		19/28 (68)	Reference	
Le, se (Lewis positive, nonsecretor)	12/12 (100)	0.89 (.79–1.01)	.24	10/12 (83)	0.81 (.57–1.17)	.32	10/12 (83)	0.81 (.57–1.17)	.32	3/11 (27)^j^	0.54 (.15–2.04)	.37	9/12 (75)	0.91 (.60–1.37)	.65
le, Se (Lewis negative, secretor)	2/2 (100)	…		0/2 (0)	…		0/2 (0)	…		0/2 (0)	…		2/2 (100)	…	
le, se^w^ (Lewis negative, weak secretor)	1/1 (100)	…		1/1 (100)	…		1/1 (100)	…		0/1 (0)	…		1/1 (100)	…	
le, se (Lewis negative, nonsecretor)	0	…		0	…		0	…		0	…		0	…	

Phenotypes were inferred from genotype type data. See Methods for descriptions of secretor and Lewis phenotypes.

Abbreviations: CI, confidence interval; IgA, immunoglobulin A; le, nonfunctional FUT3; Le, at least 1 functional gene encoding FUT3; RR, relative risk; se, nonfunctional FUT2; Se, at least 1 functional gene encoding FUT2; SNA, serum neutralizing antibody.

When vaccine take was broken down into its components of serum response and RV3-BB virus excretion, no difference was observed by secretor, Lewis, or combined Lewis and secretor status. Among secretors and nonsecretors, a serum response was detected in 21 of 32 (66%) and 11 of 13 (85%), respectively, and excretion was observed in 23 of 32 (72%) and 10 of 13 (77%), respectively. With respect to Lewis status, a serum response was detected in 29 of 40 Lewis-positive participants (73%) and 1 of 3 Lewis-negative participants (33%), whereas excretion was observed in 28 of 40 (70%) and 3 of 3 (100%), respectively. Stratification by both phenotypes combined revealed that a serum response was present in 19 of 28 Lewis-positive secretors (68%) and 10 of 12 Lewis-positive nonsecretors (83%), and excretion was found in 19 of 28 (68%) and 9 of 12 (75%), respectively. Both Lewis-negative secretors were positive for excretion but not a serum response, and the Lewis-negative weak secretor was positive for both a serum response and excretion. No difference in HBGA status was observed when serum response was separated by serum IgA and Serum Neutralising Antibody (SNA) responses. χ ^2^ analyses and RRs calculated to compare frequencies of vaccine take between HBGA groups showed no significant differences (*P* > .05 for all comparisons).

## DISCUSSION

This study demonstrated that the G3P[6] human neonatal vaccine RV3-BB produced positive cumulative vaccine take, irrespective of HBGA status. We observed no difference in positive vaccine take by secretor status, by Lewis status, or by combined Lewis and secretor status. The sample size was small, and there were only 3 Lewis-negative individuals in the study cohort, warranting cautious interpretation of these results. This is the first study to assess whether HBGA status influences take of a rotavirus vaccine based on a P[6] strain.

Importantly, Lewis positivity was not a restriction factor for the RV3-BB vaccine, with 37 of 40 Lewis-positive individuals (92.5%) and 3 of 3 Lewis-negative individuals (100%) positive vaccine take. This contrasts with observations from studies with disease-causing wild-type P[6] strains and may be a result of the intrinsic functional and structural characteristics of asymptomatic neonatal P[6] strains: RV3 has been shown to have a unique neonatal P[6] VP8*, which may be adapted to the neonatal gut to cause infection independent of HBGA status [38, 39]. In Burkina Faso, P[6] strains were observed to preferentially (but not exclusively) infect Lewis-negative children (of 27 infected children, 18 were Lewis negative, compared with 9 who were Lewis positive; odds ratio, 5.5; *P* < .0001), irrespective of secretor status [33]. Consistent with our study, secretor status has not been consistently associated with susceptibility to P[6] rotavirus infection (odds ratio, 0.4; 95% confidence interval, 0–4.1) [40]. In one study, in Swedish children, the geometric mean SNA titers to the G4P[6] ST3 strain were similar in secretors and nonsecretors [41].

Studies investigating P[8] rotavirus vaccine take and HBGA status have produced varying results, which seem to be population dependent. Epidemiological studies in the United States and France have shown nonsecretor status to be a restriction factor for P[8] rotavirus infection in infants [29, 30]. In Pakistan and Ghana, the higher rates of seroconversion to Rotarix was observed in secretors, with no difference in seroconversion based on Lewis status [25, 26]. In Nicaragua, the Le^a^ phenotype (present in Lewis-positive nonsecretors) was found to be a restriction factor to seroconversion in children after 1 dose of either Rotarix or RotaTeq [27]. In in vitro binding assays, the expressed and purified VP8* of P[8] rotaviruses bound synthetically expressed H-type 1 antigen (present in Lewis-negative secretors) and Le^b^ antigen (present in Lewis-positive secretors) but not Le^a^ antigen (present in Lewis-positive nonsecretors) [34]. In contrast, no difference in Rotarix take was observed by secretor or Lewis status in a Malawian cohort, and in Bangladesh, secretors and nonsecretors in the vaccine group of a Rotarix efficacy trial were protected similarly [28, 32]. In Tunisian infants, P[8] rotaviruses were able to infect secretors and nonsecretors, although numbers of nonsecretors in the study were low [31].

A strength of this study was that genotyping analysis was conducted using NGS, where the entire coding regions of interest for *FUT2* and *FUT3* were examined for known SNPs. Compared with methods such as PCR/restriction fragment–length polymorphism analysis, in which a limited number of specific SNPs are targeted, the “unbiased” approach used in this study gave the best possible chance of accurately designating the genotype of the individual, even without phenotypic confirmation. In this study, confirmation of Lewis and secretor genotypes by phenotypic analysis of saliva or red blood cells was not possible, as these samples were not collected as part of the vaccine trial. However, the genotypic analysis of samples collected from neonates and infants is more reliable than phenotypic analysis alone [42]. Furthermore, HBGA phenotype can vary with developmental stage, and while there is concordance of genotype and salivary phenotype, phenotyping of red blood cells may be problematic [43, 44].

This study provides evidence that the P[6]-based RV3-BB vaccine induces take, irrespective of HBGA status. This vaccine could address suboptimal efficacy of the P[8]-based vaccines in regions where the burden of P[6] rotavirus disease is high, regardless of the HGBA phenotype profile of the population. Extrapolation of results of this study to different settings requires further study. Such studies are underway in Indonesia and Malawi.

This study has some limitations. It was conducted in a relatively small sample size in a homogenous population with a small number of Lewis-negative and secretor-negative participants. The high rate of vaccine take (>90%) in this population limited the ability to explore the HBGA status of participants without vaccine take. Further studies are underway in Indonesia and Malawi to broaden the populations and improve sample sizes and power for statistical analyses.

In summary, we found that the human neonatal vaccine RV3-BB (G3P[6]) produced a positive cumulative vaccine take, irrespective of HBGA status. We observed no difference in positive vaccine take by secretor status, by Lewis status, or by combined Lewis and secretor status. This is the first study to assess whether HBGA status influences vaccine take following receipt of a rotavirus vaccine based on a P[6] strain. The RV3-BB vaccine has the potential to provide an improved level of protection, particularly in Africa, where the Lewis-negative phenotype is more prevalent and where P[6] rotavirus strains causing disease are endemic.

## Supplementary Data

Supplementary materials are available at *The Journal of Infectious Diseases* online. Consisting of data provided by the authors to benefit the reader, the posted materials are not copyedited and are the sole responsibility of the authors, so questions or comments should be addressed to the corresponding author.

jiz333_suppl_Supplementary_Table_S1

jiz333_suppl_Supplementary_Table_S2

jiz333_suppl_Supplementary_Table_S3

jiz333_suppl_Supplementary_Table_S4

jiz333_suppl_Supplementary_Table_S5

## Notes

***Acknowledgments.*** We thank the participants and their families, for taking part in this study; the midwives and antenatal staff at the Dunedin Hospital, for facilitating invitation into the study; Barry Taylor and Pam Jackson, as well as the study staff from both the Department of Women’s and Children’s Health, Dunedin School of Medicine, University of Otago, New Zealand, and the RV3 Rotavirus Vaccine Program, Murdoch Children’s Research Institute (MCRI), Victoria, Australia; Amanda Handley and Fran Justice from MCRI, for help with data management; and the Centre for Genomics Medicine—Sequencing Service and Development Platform, Victorian Clinical Genetic Services/MCRI, for their assistance.

***Financial support.*** This work was supported by the Australian National Health and Medical Research Council (project grant ID491239), the New Zealand Health Research Council International Investment Opportunities Fund Trans-Tasman Clinical Trials Collaborative Initiative (08_T02), and the Victorian Government’s Operational Infrastructure Support Program.

***Potential conflicts of interest.*** J. E. B is director of the Australian Rotavirus Surveillance Program, which is supported by research grants from GlaxoSmithKline, as well as the Commonwealth Department of Health and Aging. C. D. K. is currently employed as Senior Program Officer, Enteric and Diarrheal Disease, Bill and Melinda Gates Foundation. D. C. is currently employed by ViiV Healthcare: all work on this project was completed while at the Murdoch Children’s Research Institute. C. D. K. and Murdoch Children’s Research Institute hold a provisional patent for the RV3-BB vaccine. All authors: No reported conflicts.
