# Supplementary material for: Human Neonatal Rotavirus Vaccine (RV3-BB) Produces Vaccine Take Irrespective of Histo-Blood Group Antigen Status
Source: J Infect Dis. 2019 Nov 25;221(7):1070–8. doi: 10.1093/infdis/jiz333 (PMC7075413; doi:10.1093/infdis/jiz333)
Supplement: jiz333_suppl_Supplementary_Table_S1 [file jiz333_suppl_supplementary_table_s1.pdf]

Supplementary Table S1. Demographic characteristics

	Study cohort (n=46)
**Age* (days)**	3.0 (1.6)
**Sex (male)**	29 (63%)
**Race**	
Maori	3 (6%)
Caucasian	32 (70%)
Other**	11 (24%)
**Gestational age (weeks)**	39.41 (1.11)
**Birthweight (kg)**	3.7 (0.5)

Data are n (%) or mean (SD)

* Age at first dose of investigational product.
Calculated as (Date of Randomisation - Date of Birth)

**Pacific Islander (n=4), Asian (n=1), Maori Caucasian (n=1),
Samoan, Maori & Caucasian (n=1), Maori & Pacific Islander (n=1),
Greek, Lebanese, Caucasian (n=1), NZ/Samoan (n=1), NZ European (n=1)
