# Supplementary material for: Human Neonatal Rotavirus Vaccine (RV3-BB) Produces Vaccine Take Irrespective of Histo-Blood Group Antigen Status
Source: J Infect Dis. 2019 Nov 25;221(7):1070–8. doi: 10.1093/infdis/jiz333 (PMC7075413; doi:10.1093/infdis/jiz333)
Supplement: jiz333_suppl_Supplementary_Table_S2 [file jiz333_suppl_supplementary_table_s2.pdf]

Supplementary Table S2. FUT2 Distribution of SNPs and the allele frequencies

			SNP			Allele	
			Wild-type	Heterozygous mutant	Homozygous mutant	Primary allele	Variant allele
**Nucleotide position***	**SNP reference**	**Variant type**	**n (%)**	**n (%)**	**n (%)**	**n (%)**	**n (%)**
**171 (204)**	**rs492602**	**Synonymous**	AA<br>18 (39)	AG<br>15 (33)	GG<br>13 (28)	A<br>51 (55)	G<br>41 (45)
**216 (249)**	**rs681343**	**Synonymous**	CC<br>18 (39)	CT<br>15 (33)	TT<br>13 (28)	C<br>51 (55)	T<br>41 (45)
**357 (390)**	**rs281377**	**Synonymous**	CC<br>17 (37)	CT<br>16 (35)	TT<br>13 (28)	C<br>50 (54)	T<br>42 (46)
**385 (418)**	**rs1047781**	**Missense**	AA<br>41 (89)	AT<br>4 (9)	TT<br>1 (2)	A<br>86 (93)	T<br>6 (7)
**404 (437)**	**rs781148116**	**Missense**	GG<br>45 (98)	GA<br>1 (2)	AA<br>0 (-)	G<br>91 (99)	A<br>1 (1)
**428 (461)**	**rs601338**	**Nonsense**	GG<br>18 (39)	GA<br>15 (33)	AA<br>13 (28)	G<br>51 (55)	A<br>41 (45)
**480 (513)**	**rs1800027**	**Synonymous**	CC<br>43 (94)	CT<br>3 (6)	TT<br>0 (-)	C<br>89 (97)	T<br>3 (3)
**571 (604)**	**rs1800028**	**Nonsense**	CC<br>45 (98)	CT<br>1 (2)	TT<br>0 (-)	C<br>91 (99)	T<br>1 (1)
**739 (772)**	**rs602662**	**Missense**	GG<br>17 (37)	GA<br>16 (35)	AA<br>13 (28)	G<br>50 (54)	A<br>42 (46)
**960 (993)**	**rs485186**	**Synonymous**	AA<br>17 (37)	AG<br>16 (35)	GG<br>13 (28)	A<br>50 (54)	G<br>42 (46)

Abbreviations: SNP, Single nucleotide polymorphism

Homozygous mutant likely leading to null phenotype, that has been identified in the cohort

* A 33 base pair discrepancy was consistently observed between the nucleotide location of each SNP in the coding sequence of FUT2 between results obtained here using human reference genome assembly GRCh38 and the SNP locations published to date, though SNP references were identical.
