# Supplementary material for: Human Neonatal Rotavirus Vaccine (RV3-BB) Produces Vaccine Take Irrespective of Histo-Blood Group Antigen Status
Source: J Infect Dis. 2019 Nov 25;221(7):1070–8. doi: 10.1093/infdis/jiz333 (PMC7075413; doi:10.1093/infdis/jiz333)
Supplement: jiz333_suppl_Supplementary_Table_S3 [file jiz333_suppl_supplementary_table_s3.pdf]

Supplementary Table S3. FUT2- Genotypes & Phenotype Designation (Forward strand) Per participant

SNP	A171G	C216T	C357T	A385T	G404A	G428A	C480T	C571T	G739A	A960G	
**Participant**	**rs492602**	**rs681343**	**rs281377**	**rs1047781**	**rs781148116**	**rs601338**	**rs1800027**	**rs1800028**	**rs602662**	**rs485186**	**Phenotype (designated from genotype)**
**1**	AA	CC	TT	AA	GG	GG	CC	CC	GG	AA	Se/Secretor
**2**	GG	TT	CC	AA	GG	AA	CC	CC	AA	GG	**se/non-secretor**
**3**	AA	CC	CT	AA	GG	GG	CC	CC	GG	AA	Se/Secretor
**4**	GG	TT	CC	AA	GG	AA	CC	CC	AA	GG	**se/non-secretor**
**5**	AG	CT	CT	AA	GG	GA	CC	CC	GA	AG	Se/Secretor
**6**	AG	CT	CT	AA	GG	GA	CC	CC	GA	AG	Se/Secretor
**7**	AG	CT	CC	AA	GG	GA	CC	CC	GA	AG	Se/Secretor
**8**	GG	TT	CC	AA	GG	AA	CC	CC	AA	GG	**se/non-secretor**
**9**	AA	CC	TT	AA	GG	GG	CC	CC	GG	AA	Se/Secretor
**10**	AA	CC	TT	AA	GG	GG	CC	CC	GG	AA	Se/Secretor
**11**	AA	CC	TT	AA	GG	GG	CC	CC	GA	AG	Se/Secretor
**12**	AG	CT	CT	AA	GG	GA	CC	CC	GA	AG	Se/Secretor
**13**	AA	CC	CC	AA	GG	GG	CC	CC	GG	AA	Se/Secretor
**14**	AG	CT	CC	AA	GG	GA	CC	CC	GA	AG	Se/Secretor
**15**	GG	TT	CC	AA	GG	AA	CC	CC	AA	GG	**se/non-secretor**
**16**	GG	TT	CC	AA	GG	AA	CC	CC	AA	GG	**se/non-secretor**
**17**	GG	TT	CC	AA	GG	AA	CC	CC	AA	GG	**se/non-secretor**
**18**	AA	CC	TT	AA	GG	GG	CC	CC	GG	AA	Se/Secretor
**19**	AA	CC	CT	AA	GG	GG	CC	CC	GG	AA	Se/Secretor
**20**	GG	TT	CC	AA	GG	AA	CC	CC	AA	GG	**se/non-secretor**
**21**	AA	CC	CT	AA	GG	GG	CC	CT	GG	AA	Se/Secretor
**22**	AA	CC	TT	AA	GG	GG	CT	CC	GG	AA	Se/Secretor
**23**	GG	TT	CC	AA	GG	AA	CC	CC	AA	GG	**se/non-secretor**
**24**	AA	CC	TT	AA	GG	GG	CT	CC	GG	AA	Se/Secretor
**25**	GG	TT	CC	AA	GG	AA	CC	CC	AA	GG	**se/non-secretor**
**26**	GG	TT	CC	AA	GG	AA	CC	CC	AA	GG	**se/non-secretor**

**27**	AA	CC	TT	AA	GG	GG	CC	CC	GG	AA	Se/Secretor
**28**	AA	CC	TT	AA	GG	GG	CC	CC	GG	AA	Se/Secretor
**29**	AA	CC	TT	AA	GA	GG	CC	CC	GG	AA	Se/Secretor
**30**	AA	CC	TT	TT	GG	GG	CC	CC	GG	AA	**se/weak-secretor**
**31**	AG	CT	CT	AA	GG	GA	CT	CC	GA	AG	Se/Secretor
**32**	GG	TT	CC	AA	GG	AA	CC	CC	AA	GG	**se/non-secretor**
**33**	AG	CT	CT	AA	GG	GA	CC	CC	GA	AG	Se/Secretor
**34**	GG	TT	CC	AA	GG	AA	CC	CC	AA	GG	**se/non-secretor**
**35**	AG	CT	CT	AT	GG	GA	CC	CC	GA	AG	Se/Secretor
**36**	AG	CT	CT	AA	GG	GA	CC	CC	GA	AG	Se/Secretor
**37**	AA	CC	CT	AT	GG	GG	CC	CC	GG	AA	Se/Secretor
**38**	GG	TT	CC	AA	GG	AA	CC	CC	AA	GG	**se/non-secretor**
**39**	AG	CT	CT	AA	GG	GA	CC	CC	GA	AG	Se/Secretor
**40**	AA	CC	TT	AA	GG	GG	CC	CC	GG	AA	Se/Secretor
**41**	AG	CT	CT	AT	GG	GA	CC	CC	GA	AG	Se/Secretor
**42**	AG	CT	CT	AT	GG	GA	CC	CC	GA	AG	Se/Secretor
**43**	AA	CC	TT	AA	GG	GG	CC	CC	GG	AA	Se/Secretor
**44**	AG	CT	CT	AA	GG	GA	CC	CC	GA	AG	Se/Secretor
**45**	AG	CT	CT	AA	GG	GA	CC	CC	GA	AG	Se/Secretor
**46**	AG	CT	CC	AA	GG	GA	CC	CC	GA	AG	Se/Secretor

Abbreviations: SNP, Single Nucleotide Polymorphism

KEY

Homozygous Dominant

Heterozygous

Homozygous Recessive- Synonymous

Homozygous Recessive- Stop/Nonsense

Homozygous Recessive- Missense, Weak

Homozygous Recessive- Missense, Probably Damaging
