# Supplementary material for: Human Neonatal Rotavirus Vaccine (RV3-BB) Produces Vaccine Take Irrespective of Histo-Blood Group Antigen Status
Source: J Infect Dis. 2019 Nov 25;221(7):1070–8. doi: 10.1093/infdis/jiz333 (PMC7075413; doi:10.1093/infdis/jiz333)
Supplement: jiz333_suppl_Supplementary_Table_S4 [file jiz333_suppl_supplementary_table_s4.pdf]

Supplementary Table S4. FUT3 Distribution of SNPs and the allele frequencies

			SNP (reverse strand)			Allele	
			Wild-type	Heterozygous mutant	Homozygous mutant	Primary allele	Variant allele
**Nucleotide position**	**SNP reference**	**Variant type**	**n (%)**	**n (%)**	**n (%)**	**n (%)**	**n (%)**
**47**	**rs145362171**	**Missense**	GG<br>42 (98)	GC<br>1 (2)	CC<br>0 (-)	G<br>85 (99)	C<br>1 (1)
**59**	**rs28362459**	**Missense (reducing)**	TT<br>35 (81)	TG<br>6 (14)	GG<br>2 (5)	T<br>76 (88)	G<br>10 (12)
**93**	**rs757125324**	**Synonymous**	CC<br>42 (98)	CT<br>1 (2)	TT<br>0 (-)	C<br>85 (99)	T<br>1 (1)
**202**	**rs812936**	**Missense**	TT<br>34 (79)	TC<br>8 (19)	CC<br>1 (2)	T<br>76 (88)	C<br>10 (12)
**273**	**rs146514727**	**Synonymous**	CC<br>42 (98)	CT<br>1 (2)	TT<br>0 (-)	C<br>85 (99)	T<br>1 (1)
**314**	**rs778986**	**Missense**	CC<br>35 (81)	CT<br>7 (17)	TT<br>1 (2)	C<br>77 (90)	T<br>9 (10)
**508**	**rs3745635**	**Missense**	GG<br>40 (93)	GA<br>2 (5)	AA<br>1 (2)	G<br>82 (95)	A<br>4 (5)
**1067**	**rs3894326**	**Missense**	TT<br>37 (86)	TA<br>5 (12)	AA<br>1 (2)	T<br>79 (92)	A<br>7 (8)

Abbreviations: SNP, Single Nucleotide Polymorphism

(grey shading) Homozygous mutant likely leading to null phenotype, that has been identified in cohort
