# Supplementary material for: Human Neonatal Rotavirus Vaccine (RV3-BB) Produces Vaccine Take Irrespective of Histo-Blood Group Antigen Status
Source: J Infect Dis. 2019 Nov 25;221(7):1070–8. doi: 10.1093/infdis/jiz333 (PMC7075413; doi:10.1093/infdis/jiz333)
Supplement: jiz333_suppl_Supplementary_Table_S5 [file jiz333_suppl_supplementary_table_s5.pdf]

Supplementary Table S5. FUT3- Genotypes & Phenotype Designation (Forward strand)- Per participant

SNP	T1067A	G508A	C314T	C273T	T202C	C93T	T59G	G47C	
Participant	rs3894326	rs3745635	rs778986	rs146514727	rs812936	rs757125324	rs28362459	rs145362171	Phenotype (designated from genotype)
1	AA	CC	GG	GG	AA	GG	AA	CC	Le/Lewis positive
2	AA	CC	GG	GG	AA	GG	AA	CC	Le/Lewis positive
3	AA	CC	GG	GG	AA	GG	AA	CC	Le/Lewis positive
4	AA	CC	AG	GG	GA	GG	AA	CC	Le/Lewis positive
5	AT	CC	GG	GG	GA	GG	AC	CC	Le/Lewis positive
6	AA	CC	GG	GG	AA	GG	AA	CC	Le/Lewis positive
7	AA	CC	GG	GG	AA	GG	AA	CC	Le/Lewis positive
8	AA	CC	GG	GG	AA	GG	AA	CC	Le/Lewis positive
9	AA	CC	AG	GA	GA	GG	AA	CC	Le/Lewis positive
10	AA	CC	GG	GG	AA	GG	AA	CC	Le/Lewis positive
11	AA	CC	GG	GG	AA	GG	AA	CC	Le/Lewis positive
12	AA	CC	AA	GG	GG	GG	AA	CG	**le/Lewis negative**
13	AA	CC	GG	GG	AA	GA	AA	CC	Le/Lewis positive
14	AA	CC	AG	GG	GA	GG	AA	CC	Le/Lewis positive
15	AA	CC	GG	GG	AA	GG	AA	CC	Le/Lewis positive
16	AA	CC	GG	GG	AA	GG	AA	CC	Le/Lewis positive
17	AA	CC	AG	GG	GA	GG	AA	CC	Le/Lewis positive
18	AA	CC	GG	GG	AA	GG	AA	CC	Le/Lewis positive
19	AT	CC	GG	GG	GA	GG	AC	CC	Le/Lewis positive
20	AA	CC	GG	GG	AA	GG	AA	CC	Le/Lewis positive
21	AA	CC	GG	GG	AA	GG	AA	CC	Le/Lewis positive
22	Not determined								
23	Not determined								
24	AA	CT	GG	GG	AA	GG	AC	CC	Le/Lewis positive
25	AA	CC	GG	GG	AA	GG	AA	CC	Le/Lewis positive

**26**	AA	CT	AG	GG	GA	GG	AC	CC	Le/Lewis positive
**27**	AA	CC	GG	GG	AA	GG	AA	CC	Le/Lewis positive
**28**	TT	CC	GG	GG	AA	GG	CC	CC	**le/Lewis negative**
**29**	AT	CC	GG	GG	AA	GG	AC	CC	Le/Lewis positive
**30**	AA	TT	GG	GG	AA	GG	CC	CC	**le/Lewis negative**
**31**	AA	CC	GG	GG	AA	GG	AA	CC	Le/Lewis positive
**32**	AA	CC	AG	GG	GA	GG	AA	CC	Le/Lewis positive
**33**	AT	CC	GG	GG	AA	GG	AC	CC	Le/Lewis positive
**34**	AA	CC	GG	GG	AA	GG	AA	CC	Le/Lewis positive
**35**	AA	CC	GG	GG	AA	GG	AA	CC	Le/Lewis positive
**36**	AA	CC	GG	GG	AA	GG	AA	CC	Le/Lewis positive
**37**	Not determined								
**38**	AA	CC	AG	GG	AA	GG	AA	CC	Le/Lewis positive
**39**	AT	CC	GG	GG	AA	GG	AA	CC	Le/Lewis positive
**40**	AA	CC	GG	GG	AA	GG	AA	CC	Le/Lewis positive
**41**	AA	CC	GG	GG	AA	GG	AA	CC	Le/Lewis positive
**42**	AA	CC	GG	GG	AA	GG	AA	CC	Le/Lewis positive
**43**	AA	CC	GG	GG	AA	GG	AA	CC	Le/Lewis positive
**44**	AA	CC	GG	GG	AA	GG	AA	CC	Le/Lewis positive
**45**	AA	CC	GG	GG	AA	GG	AA	CC	Le/Lewis positive
**46**	AA	CC	GG	GG	AA	GG	AA	CC	Le/Lewis positive

Abbreviations: SNP, Single Nucleotide Polymorphism

KEY

Homozygous Dominant

Heterozygous

Homozygous Recessive- Missense, Reducing

Homozygous Recessive- Missense, Inactivating
